# Key themes and approaches in palliative and end-of-life care education for the general public: a systematic review

**DOI:** 10.1186/s12904-025-01852-w

**Published:** 2025-08-07

**Authors:** Muzeyyen Seckin, Rumandeep Tiwana, David Fry, Cara Bailey

**Affiliations:** 1 Department of Nursing and Midwifery, School of Health Sciences, College of Medicine and Health, University of Birmingham, East Wing, Medical School, Edgbaston, Birmingham, B15 2TT UK; 2 NHS Birmingham and Solihull ICB, Birmingham, UK; 3 St Giles Hospice, Staffordshire, UK

**Keywords:** Palliative care, End of life care, General public/Citizens, Education or training

## Abstract

**Background:**

Families, friends, and communities play a vital role in supporting individuals facing declining health, caregiving duties, loss, or grief, especially with the growing desire to die at home. The general public can significantly impact end-of-life care and offer essential support mechanisms. This review aimed to explore and identify key educational components related to palliative and end-of-life care for citizens, volunteers, and the general public.

**Methods:**

A mixed-method systematic review was conducted, incorporating four electronic databases (MEDLINE, PsycINFO, CINAHL, and the Cochrane Library) and grey literature searches, and quality was assessed using Hawker et al.’s (2002) critical appraisal checklists.

**Results:**

Twenty studies published between 2011 and 2023 were included, covering topics in palliative, end-of-life care, and bereavement education. These studies involved a total of 10,307 participants and identified 16 different educational programmes for the public, volunteers, and lay caregivers. The analysis revealed six main themes: foundational concepts and philosophies, communication and decision-making, planning and preparation, symptom management, end-of-life care practices, and caregiving support.

**Conclusions:**

This review highlights the importance of training programmes to improve community involvement in caregiving and enhance the quality of care for individuals with life-limiting conditions. Expanding access to such educational resources can empower more people to contribute confidently to end-of-life care in their communities.

**PROSPERO-ID:**

CRD42024533124

**Supplementary Information:**

The online version contains supplementary material available at 10.1186/s12904-025-01852-w.

## Introduction

Palliative and end-of-life care (PEoLC) positively impacts the quality of life for both children and adult patients with advanced life-threatening illnesses, as well as their families, by providing timely identification and management of their physical, psychological, social and spiritual problems [1]. By 2060, it is estimated that 48 million people will die from life-limiting health conditions, accounting for 47% of all global deaths [2]. This means that the number of individuals requiring palliative care will double over the next four decades worldwide [3]. With the increasing demand for palliative care services, families, volunteers, and communities can provide informal care and support to individuals facing serious illness [4–6].

Palliative care as a public health strategy offers the best approach that is accessible to everyone in the community when needed [7]. Public health palliative care education programmes are crucial for enhancing public knowledge [8, 9] and providing training for community-based volunteers to support communities in responding to palliative, end-of-life (last year of life [10]), and bereavement needs [4, 11]. A previously published scoping review highlighted the strong need for targeted palliative care educational interventions to improve public knowledge and awareness of palliative care [12]. These education programmes are increasingly recognised as essential interventions to improve the accessibility and effectiveness of palliative and end-of-life care for individuals and their families [9, 13]. The aim of these programmes is typically to provide essential knowledge on palliative and end-of-life care practices, including managing distressing symptoms, communication, advance care planning, and caregiving to non-professional caregivers, hospice or community volunteers and the general public [5, 14, 15].

Recently, many countries have developed new public health palliative care education courses aimed at enabling individuals to support and assist their family, friends, and community members with palliative, end-of-life, and bereavement care. Examples include the Last Aid Course, now available in 20 countries and led by Georg Bollig [8, 16], the End-of-Life Aid Skills for Everyone (EASE) programme [17] in Scotland, and community-based hospice palliative care volunteers in Canada [15]. Despite the growing interest in public health education for palliative and end-of-life care, there remains a gap in systematically identifying the core components of these programmes and evaluating their long-term sustainability and integration into existing healthcare systems. Therefore, this systematic review was conducted to evaluate and identify community-based palliative and end-of-life care education, and training programmes and resources aimed at supporting individuals with advanced life-limiting illnesses or palliative care needs. It also aims to assess the feasibility, acceptability, and potential effectiveness of these community-based education and training programmes and resources in care provision.

## Methods

### Research aims/questions

To systematically explore and identify the essential educational or training components relating to palliative and end-of-life care for citizens, volunteers, or the general public, with the ultimate aim of improving the quality of care and support available to individuals and their families facing advanced life-limiting illnesses or palliative care needs.

Main research question: What palliative and end-of-life care educational programmes and resources are currently available or provided to citizens and volunteers?

Main objectives of the review regarding main research question:

- To identify community-based palliative and end-of-life care education and training components.
- To assess the feasibility, acceptability and potential effectiveness of these programmes and resources in care provision.

### Design and protocol registration

This is a mixed-methods systematic review guided by the Joanna Briggs Institute (JBI) Methodology for mixed-methods systematic reviews [18] and the Preferred Reporting Items for Systematic Review and Meta-Analyses (PRISMA) [19]. The protocol of this systematic review was registered in PROSPERO [CRD42024533124] and is available online at https://www.crd.york.ac.uk/prospero/display_record.php?RecordID=533124. This review was grounded in a critical realist framework [20] to explore the educational components and delivery methods of palliative and end-of-life care for the general public. It systematically examines various aspects of palliative and end-of-life care education for citizens.

This review included four electronic databases (MEDLINE, PsycINFO, CINAHL, and the Cochrane Library) and grey literature (including Google, Google Scholar, and national/international websites related to palliative and end-of-life care education for the public or citizens). Relevant MeSH terms (“palliative care”, “end of life care”, “education/training” and “citizen or public”) and their synonyms were used to identify pertinent studies for this review between February and April 2024 (for the full search strategy in MEDLINE, see Supplementary Material Table 1). MS developed the search strategies, which were double-checked by a librarian to ensure consistency across all databases. Only primary research studies focusing on palliative and end-of-life care education, or training targeted at the general public, citizens, or volunteers from the public were included in this review. To capture current educational content, studies published within the last ten years (2013–2024) were included, and only English-language studies were considered.

**Table 1:** Inclusion and exclusion criteria

Inclusion criteria	Exclusion criteria
**Population**	
Age of participants ≥ 16 years Citizens and public, (community) volunteers	Participants < 16 years old Healthcare professionals, students in healthcare, patients, and caregivers.
**Context**	
Palliative and end of life care	Not in the context of palliative and end of life care
**Study design**	
Primary studies including qualitative, quantitative, and mixed-methods	Case studies, systematic and literature reviews, unpublished studies, conference papers/abstracts, editorial papers/letters, books, dissertations, and commissioned reviews.
**Outcomes**	
Education or/and training contents for palliative and end of life care	Not included education/training contents/resources for citizens, public and community volunteers.
**Others**	
English language	Non-English language
Last 10 years (2013–2024)	Before 2013

### Inclusion and exclusion criteria

Primary (empirical) studies were included if they involved the general public, citizens, (community) volunteers, or families/friends/neighbours interested in palliative and end-of-life care education or information to support individuals with advanced life-limiting illnesses or palliative care needs, or if they aimed to personally improve their palliative and end-of-life care knowledge for the future (Table 1).

Studies were excluded if they were secondary studies including literature reviews, systematic reviews, scoping reviews, and other types of evidence syntheses, involved healthcare providers (including nurses, doctors, and academics) or healthcare students as course participants, or focused on patients and their caregivers (particularly formal or paid or informal caregivers) (Table 1). Any studies that did not include palliative and end-of-life care education or training for the general public, citizens, or community volunteers were excluded. Other types of studies and documents, including policies, reviews, conference abstracts, unpublished works, books, book chapters, theses, etc., were also excluded.

### Screening and data extraction

The search results were imported into Covidence [21], an online tool for systematic review management, which performs the initial screening and exclusion of duplicate records. Studies were evaluated based on the inclusion and exclusion criteria by MS and double-checked by CB. Following the full-text screening stage, relevant studies were identified by MS and CB. A data abstraction form developed by MS and CB was used to capture the characteristics of studies in relation to the review questions/objectives. MS and RT divided the identified studies for data extraction, which was done independently. MS then checked and compared the data to finalise the results. The Covidence software [21] programme was used to remove duplicates, screen studies, extract data, and assess the quality of studies.

### Quality assessment

The quality of the studies was assessed using an appraisal tool developed by Hawker, Payne [22] to evaluate quantitative, qualitative, and mixed-methods studies. This tool uses a checklist to assess study quality and provides a summed score (ranging from 40 (good) to 10 (very poor)). Half of the studies were assessed independently by RT, and with the remaining studies assessed by MS. MS then reviewed the final data and discussed any inconsistencies with RT and CB before reaching a conclusion. It provides a thorough framework for evaluating the quality of both quantitative and qualitative studies in systematic reviews. This tool enables a systematic assessment of methodological rigor, ensuring that the studies included are sound and dependable, which ultimately enhances the reliability of the review’s conclusions. Only one study was rated as having fair quality compared to other studies. On the basis of the quality assessment, none of the studies were excluded. The results of the critical appraisal are presented in Supplementary Material Table 2.

**Table 2:** Details of included studies

Details of included studies		
		**Total (** ***n*** ** = 20)**
**Summarised articles characteristics. (n) represents the number of papers with the characteristic**		
Country^#^ (in which the study conducted)	Canada, n (%)	7 (35)
	United Kingdom, n (%)	5 (25)
	Australia, n (%)	4 (20)
	United States of America, n (%)	4 (20)
	Germany, n (%)	2 (10)
	New Zealand, n (%)	2 (10)
	Austria, n (%)	1 (5)
	Brazil, n (%)	1 (5)
	Italy, n (%)	1 (5)
	Japan, n (%)	1 (5)
	Hong Kong, n (%)	1 (5)
	South Korea, n (%)	1 (5)
	Switzerland, n (%)	1 (5)
Date of publication	2011–2014, n (%)	4 (20)
	2017–2019, n (%)	3 (15)
	2020–2023, n (%)	14 (70)
Study design	Mixed-method, n (%)	10 (50)
	Pre-post design, n (%)	4 (20)
	Survey design	3 (15)
	Qualitative, n (%)	2 (10)
	Multi-method	1 (5)
Phase of care considered within the studies for education	Palliative, n (%)	7 (35)
	End of life, n (%)	6 (30)
	Palliative and end of life, n (%)	6 (30)
	Palliative, end of life and bereavement, n (%)	1 (5)
Population description	Volunteers, n (%)	13 (65)
	Public, n (%)	6 (30)
	High school student, n (%)	1 (5)
Research population	Total number of participants, n (%)	10,307(100)
	Public, n (%)	7447 (72)
	Volunteers, n (%)	2617 (25)
	Patients, n (%)	140 (1)
	Healthcare providers, n (%)	102 (1)
**Summarised patients’ descriptive for included 20 articles. (n) represented the number of relevant participants (** ***n*** ** = 10065)**		
Biological sex***	Total number of participants	8498
	Female, n (%)	7109 (84)
	Male, n (%)	1389 (16)
Age****	Age range	17–80+
	Mainly age range in the studies	40–65

*** 4 studies did not report biological sex of the participants

**** 5 studies did not report mean age or age range of the participants

### Synthesis of the results

After data extraction, the core themes were descriptively synthesised by MS using a Microsoft Excel sheet, addressing the research objectives. Thematic analysis was used to evaluate and identify palliative and end of life care education components for the general public/citizens and community volunteers [23]. Codes were generated from education content that had been used in the studies, and these codes were then grouped. After creating initial themes and subthemes, the candidate themes were discussed with the team and finalised with their narrative explanations to identify the central concepts of palliative and end-of-life care education for the general public. Microsoft Excel was also used to analyse basic descriptive statistics for the studies and population characteristics. After identifying the main themes of the educational programmes, a final framework was developed to highlight the key educational points for the general public and community volunteers. This is presented as the Palliative and End-of-Life Care Education Framework, targeting the general public and community volunteers, in this review.

### Outcomes

Studies were analysed based on the following outcomes:

- Identification of palliative and end-of-life care education or training models or resources. This includes identifying the education or training topics or content, education materials, structure or format and delivery methods.
- Identification of their effectiveness and acceptability by users.
- Understanding the specific roles played by healthcare providers, community organisations, and other stakeholders in delivering this education to citizens.

## Results

### Study selection

**Fig. 1:**
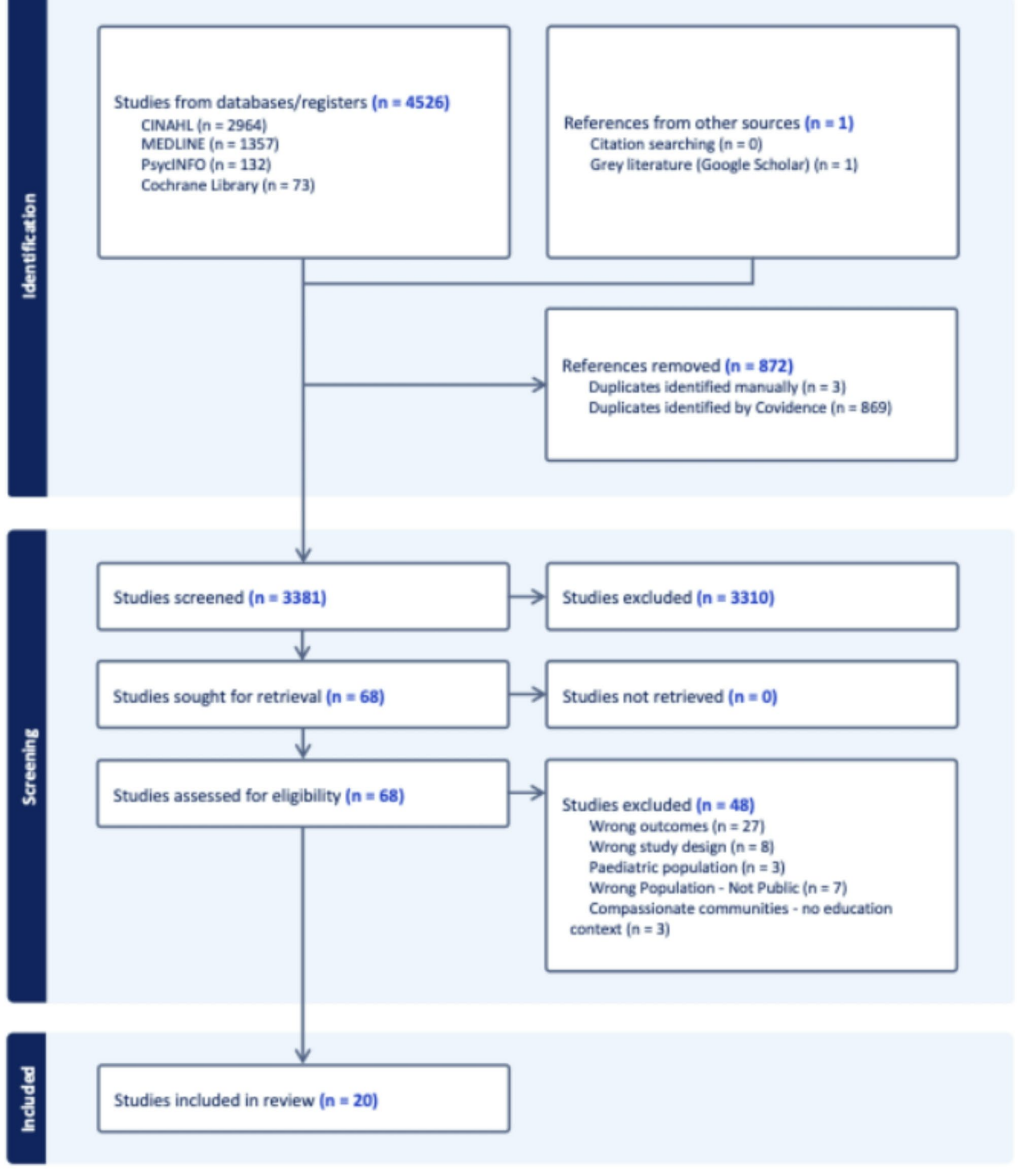
PRISMA flow chart generated from the Covidence (2024) showing the search results and study selection process

After conducting database and grey literature searches, a total of 4,527 papers were imported into Covidence [21]. Following deduplication, 3,381 records were screened based on title and abstract. Sixty-eight papers were selected for full-text screening, and 48 of these were excluded for not meeting the eligibility criteria. As a result, 20 studies were identified that aligned with the review’s aims and objectives (Fig. 1).

### Characteristics of included studies and study participants

Twenty studies were included in this review. The main study designs were mixed-methods (*n* = 10) followed by pre-post design (*n* = 4), survey (*n* = 3), qualitative (*n* = 2) and multi-method (*n* = 1), respectively (Table 2). The studies were conducted in 13 different countries, mainly including Canada (*n* = 7), the UK (*n* = 5), Australia (*n* = 4), and the USA (*n* = 4). Most of the studies were published between 2020 and 2023. The phase of care considered within the studies for education and training was palliative (*n* = 7) and end-of-life (*n* = 6) care, or both (*n* = 6). Although some studies included mixed populations, more than half focussed on volunteer samples for education in palliative and end-of-life care. The total number of participants in this review was 10,307, and the relevant population (after excluding patients and healthcare professionals) was 10,065. The majority of these participants were female.

### Education programmes regarding palliative and end of life care

There are various education and training programmes aimed at increasing the public’s and volunteers’ knowledge of palliative and end-of-life care (Table 3). These programmes are mainly delivered in-person, but there are also options available through online educational websites or webinars designed to raise awareness and knowledge about palliative and end-of-life care for the general public and volunteers. Education for volunteers tends to be more practice-based, as they often support individuals in hospices or at home. In particular, community connectors [4, 14] or volunteer navigators [24, 25] or peer facilitators [26] receive more extensive training to organise volunteers or community helpers to provide relevant support for those requiring palliative and end-of-life care.

**Table 3:** Education programme/resource from studies

Education programme/resource	Number of studies	Online/in-person	Who is delivering the education/training?	Follow-up duration	Stakeholder involvement	Education and training components or topics considered in the studies						Effectiveness	Acceptability
						Foundational concepts and philosophies	Importance of communication and decision-making	Planning and preparation	Symptom management and comfort	End-of-life care practices	Support and caregiving to carers		
Compassionate Communities Connectors [4, 14]	2	In-person	PC expert	15 months	Compassionate communities with health services	**√**	**√**	**√**				Volunteering improved social connections and reduced isolation.	Volunteers and families found it helpful. Volunteers felt more connected.
Massive Open Online Course, Dying2Learn [30, 36]	2	Online	PC expert	Pre-post	Care research and PC networks	**√**	**√**			**√**		Participants liked the online course and learned about death. Death knowledge improved, especially for those with experience.	Participants felt more confident about death and end-of-life.
Last Aid Course [8, 16]	2	Both	PC expert	Pre-post	N/A	**√**	**√**	**√**	**√**	**√**	**√**	Last Aid course was well-rated. Online version was easy to understand.	100% of participants would recommend the course. Participants liked the online format
Nav-CARE Navigation: Connecting, Advocating, Resourcing, Engaging [24, 25]	2	In-person	Researcher	6, 12, 18 months	Hospice and community-based organisations	**√**			**√**		**√**	Volunteers felt more confident. Improved skills, but more focus needed on advance care planning.	Users found it helpful and liked the program.
Peer Education Training Program [33]	1	In-person	Researcher	4, 6, 12–18 moths	Community organisations and support groups	**√**	**√**				**√**	Volunteers helped with end-of-life topics.	N/A
Last Watch/No Veteran Dies Alone [29]	1	Written manual/ DVDs	Researcher/ project coordinator	4 months	National Hospice and palliative leadership	**√**	**√**		**√**	**√**		Volunteers helped with care plans and emotional support.	Volunteers provided good emotional support.
Hospital Volunteers’ End-of-life Care Training [11]	1	Mixed	Researcher	Pre-post	N/A		**√**	**√**	**√**	**√**		Volunteers needed more training, especially with role-playing.	Volunteers wanted more diverse training methods
End-of-Life Aid Skills for Everyone [17]	1	Both	Facilitators	Pre-post	Partnership for PC	**√**	**√**	**√**	**√**	**√**	**√**	Successful adoption by hospices, charities, and community organizations interested in facilitating this training.	Positive feedback on the structure and practical application of the training.
Dying Education for adolescents [34]	1	In-person	PC expert	Pre-post	N/A	**√**		**√**		**√**		Adolescents learned to cope with loss.	The program helped students think about life and death.
Navigator Training (Native Patient Navigators) [27]	1	In-person	Administrator	3 months	Hospice, research and health board collaboration	**√**						20% improvement in knowledge, better results in cancer care.	Participants found the workshops helpful.
Training Program for Palliative Care Volunteers [32]	1	In-person	Researcher	10 weeks	N/A	**√**	**√**	**√**	**√**	**√**	**√**	Improved care for terminally ill patients.	N/A
End-of-life Phenomena Training Module [28]	1	In-person	Researcher	Pre-post	N/A	**√**	**√**		**√**			Volunteers felt more prepared for end-of-life care.	N/A
Community-led peer-facilitated advanced care planning education model [26]	1	Mixed	Trained community volunteers	6 and 12 months	Community-based organisations	**√**	**√**	**√**		**√**		Training improved knowledge on advance care planning.	Participants were satisfied with the course.
Namaste Care [37]	1	In-person	Researcher	3 months	N/A			**√**	**√**			‘Namaste Care’ improved mood for residents with dementia.	N/A
Volunteer Training Program on the Learning Support of Children in Hospice Palliative Care [31]	1	In-person	Researcher	Pre-post	N/A	**√**	**√**		**√**		**√**	Volunteers felt more confident in family communication.	Training was well-received.
Holistic capacity-building program for volunteers in community-based end of life care [35]	1	In-person	Researcher	6 months	Charities trust	**√**	**√**	**√**	**√**		**√**	Positive impact on self-care and end-of-life knowledge.	N/A

PC; palliative care, N/A; not applicable

Studies typically evaluated the effectiveness of these educational programmes over short time periods (mainly 3–6 months or pre- and post-education). The findings indicate that these education modules and materials are acceptable and effective in increasing knowledge and awareness of palliative and end-of-life care. Participants also reported being generally satisfied with the duration and components of the education, and found the provided education useful for improving their knowledge and promoting a dignified death [8, 11, 16, 17, 26–31]. However, the feasibility and acceptability of the care programmes depends on having a clearly identified targeted population, organisational capacity, skilful messaging and stable and engaged leadership [24]. These programmes were usually delivered by well-educated and expert healthcare providers.

Studies included stakeholders and organisational networks to reach and recruit relevant participants from their target populations [4, 14, 27, 29, 32, 33]. Sometimes these organisations were used as venues, or as platforms to educate volunteers who could support individuals with palliative and end-of-life care needs. These kinds of partnerships help them to develop a curriculum and implement their education to their target population (including public, volunteers) [24–26, 29, 33]. Some studies targeted students as their educational population, utilising universities and high schools [34]. There was collaborative work in the studies between researchers and hospitals or other healthcare organisations. Additionally, studies often used healthcare providers to deliver this education to their target populations, fostering collaborative work between clinical settings and the community.

### Education and training components or topics considered in the studies

**Fig. 2:**
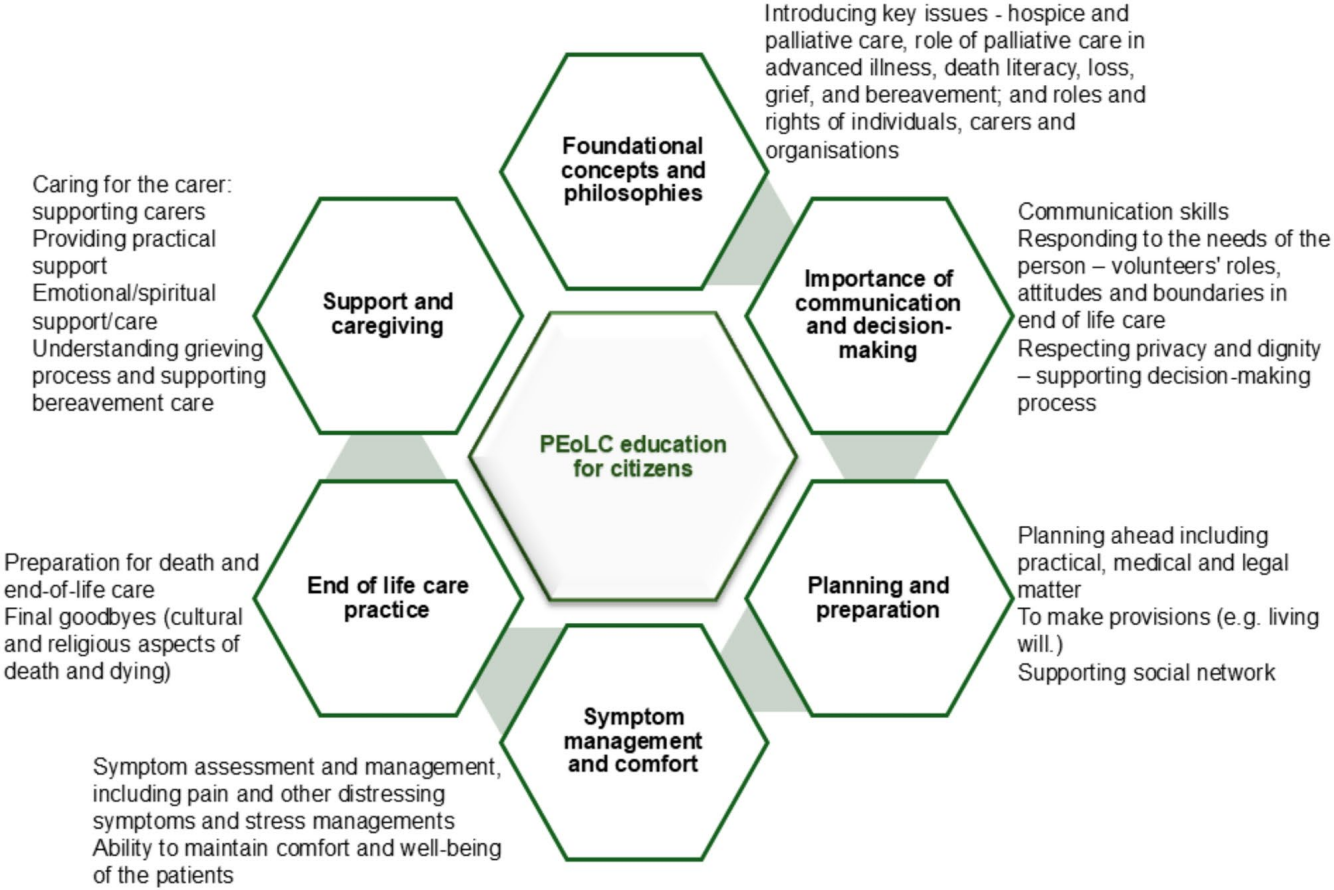
Education and training topics considered in the studies for citizens and volunteers

Six main themes were identified for education and training topics considered in the studies for public and volunteers (Fig. 2).

#### Foundational concepts and philosophies about palliative care, death, dying, and end-of-life care

##### Objective

(a) Building foundational knowledge on how palliative care supports individuals with life-limiting conditions, and (b) informing volunteers and the public of the ethical and legal principles that guide palliative and end-of-life care to ensure they act within appropriate boundaries [4, 8, 14, 16, 17, 24–29, 31–35].

Introducing key issues and covering essential information is the first educational topic in many studies. They begin by providing information about hospice and palliative care philosophies to help understand the needs of individuals requiring palliative and end-of-life care. Particularly, understanding the values and knowledge surrounding end-of-life care, as well as perspectives on loss, grief, and bereavement support, were highlighted in the studies. To effectively support individuals living with advanced, life-limiting illnesses, caregivers, volunteers, and individuals must understand their roles and rights, as well as the needs of those they are supporting. Additionally, legal and ethical issues were addressed to equip volunteers with an understanding of what they can do and where to seek further help to support these individuals.

#### Importance of communication and decision-making

##### Objective

Equip the public and volunteers with the skills to navigate sensitive conversations and support informed decision-making [4, 8, 11, 14, 16, 17, 26, 28–33, 35, 36].

The importance of effective (verbal and non-verbal) communication is highlighted in providing comfort to individuals at the end of life and in understanding the needs of those living with advanced, life-limiting illnesses. It is essential for volunteers and carers to grasp the care preferences of individuals, particularly in the decision-making process related to end-of-life care and advance care planning. Clear and compassionate communication enables volunteers to understand their roles, attitudes, and boundaries when supporting those facing end-of-life challenges.

#### Planning and preparation

##### Objective

Enable the public and volunteers to help individuals and families in planning their care and making informed choices for the future [4, 8, 11, 14, 16, 17, 26, 30, 32, 34–37].

Planning and preparation of care and advance practices were common topics in the educational or training programmes provided to volunteers and the public. Preparing individuals living with advanced, life-limiting illnesses involves making plans and decisions about their care preferences, which is highly relevant to providing personalised care for them. This included addressing practical, medical, and ethical matters, often considering the role of social networks in providing support. Also, the importance of safeguarding children and vulnerable adults were provided as an education component to the volunteers.

#### Symptom management and comfort

##### Objective

Providing knowledge on recognising and managing both physical and emotional symptoms to enhance quality of life and overall well-being [8, 11, 16, 17, 24, 25, 28, 29, 31, 32, 35, 37].

Educational programmes provided detailed guidance on symptom assessment and management, particularly in relation to pain and other distressing symptoms. Practical guidance on symptom management (pain relief, emotional support, etc.) was highlighted to maintain comfort and dignity for patients. Some studies also highlighted the importance of eating and drinking in end-of-life care. In addition to physical symptoms, programmes provided guidance on managing emotional distress and stress, emphasising the importance of maintaining comfort and well-being for both patients and their families. Medication and medication administration methods were also covered in the educational courses. Furthermore, the importance of touch and human connection (including hand and foot massage training) was reported as an educational component for families and carers.

#### End-of-life care practices

##### Objective

Preparing volunteers and community members to support individuals and families through the dying process, respecting diverse beliefs in their community [8, 11, 16, 17, 26, 29, 30, 32, 34, 36].

Preparation for death and providing end-of-life care are essential for helping individuals say their final goodbyes. This topic was considered a vital area of education for volunteers and the public. Understanding cultural and religious perspectives, rituals associated with death, and the cultural aspects of dying were crucial to the acceptance of death, making final wishes, and addressing any unfinished business. These aspects were also emphasised in the context of bereavement and self-care.

#### Support and caregiving to carers

##### Objective

Equip volunteers and the public with strategies to provide care and support not only to patients but also to their families and caregivers during palliative, end-of-life, and bereavement care [8, 16, 17, 24, 25, 31–33, 35].

Support and caregiving were less commonly addressed in educational programmes, but a few studies highlighted the importance of caring for the carer. Recent palliative and end-of-life care educational programmes have incorporated components on emotional, spiritual, practical, and self-care support for carers, recognising the significant role they play. Offering bereavement support to families and friends with an understanding of grief responses and providing ongoing support through the stages of grief and loss were some of the education topics.

## Discussion

The review identified a variety of public health palliative and end of life care education programmes aimed at general public and community volunteers. Education programmes or resources were delivered both in-person or/and online to extend the reach of palliative and end of life care education to a broader audience. The Last Aid Course initially begun as in-person programme and was later delivered online during the COVID-19 pandemic [8, 16]. Both in-person and online versions were found as acceptable and relevant to deliver the palliative and end of life care education to participants [8, 16]. Although some participants reported a preference for face-to-face education programmes [17], online programmes were particularly useful in enhancing accessibility for participants who may geographically dispersed or unable to attend in-person session [16, 38]. While online versions can reduce geographical inequalities in public health education, they may not be adequate on their own for practice-based training in roles like community connectors or volunteer navigators.

Core components of the education programmes include six main topics; (1) foundational concepts and philosophies; (2) importance of communication and decision-making; (3) planning and preparation; (4) symptom management and comfort; (5) end-of-life care practices; and (6) support and caregiving. When considering palliative, end-of-life and bereavement care as a whole, these topics consisted of all phases of care faced by individuals with advanced life-limiting illness or conditions and their families. Plain-language version of these education components provided to general public and community volunteers. However, the challenge is to develop palliative and end of life care comprehensive and systematic information on these topics at the population level [3]. Based on the targeted population with the different education level, education contents can be different. Additionally, supporting caregivers and families’ needs should be considered when any educational outline is drafted in any community, as they are a crucial part of the sustainability of palliative care for patients and, after their death, their own health and well-being as part of public health strategies. This will be included in both formal education for healthcare providers, as well as informal education for the general public and volunteers.

The involvement of healthcare providers, community organisation and other stakeholders was identified as a critical factor in the delivery of palliative and end-of-life care education. Especially, nurses and palliative care specialist led these programmes, ensuring the content was evidence-based and tailored to the needs of informal carers and volunteers. Community organisations provided help in coordinating of these programmes with providing place and advertising the programmes [7]. These community organisations are important to support and provide the support and help for palliative and end of life care education for general public [7]. This kind of collaborative approach allowed the integration of thus education into the existing community networks and fostering a more supportive environment for educators, participants, and researchers [6]. Similar findings can be seen in a recent review by Ontrakrai, Bailey [39], which scoped education programmes for volunteers caring for children and young people, highlighting the strength of community engagement.

Although, the education programmes and resources were identified as acceptable and useful for enhancing the public’s knowledge of palliative and end-of-life care, a significant gap in the studies was the limited evaluation of long-term outcomes and actual effects on providing support in individuals’ palliative and end of life needs. The studies provided positive improvement for increased knowledge, confidence and satisfaction of the participants regarding the understanding of palliative and end-of-life care. However, there is limited evidence on how these improvements influence or lead to sustained behavioural changes among individuals receiving palliative care in the community and their families. An evaluation of the long-term effects or impacts of these training programmes on palliative and end-of-life care recipients is needed in practice, especially in terms of reducing the burden on healthcare systems and families. As reported by Collins, Brown [40], more empirical work is needed to support the reorientation of health services to improve end-of-life care, as there is currently limited evidence on the impact of public health palliative care in improving end-of-life healthcare utilisation outcomes. This reorientation is essential to achieve system-wide change and ensure quality end-of-life care for all.

**Fig. 3:**
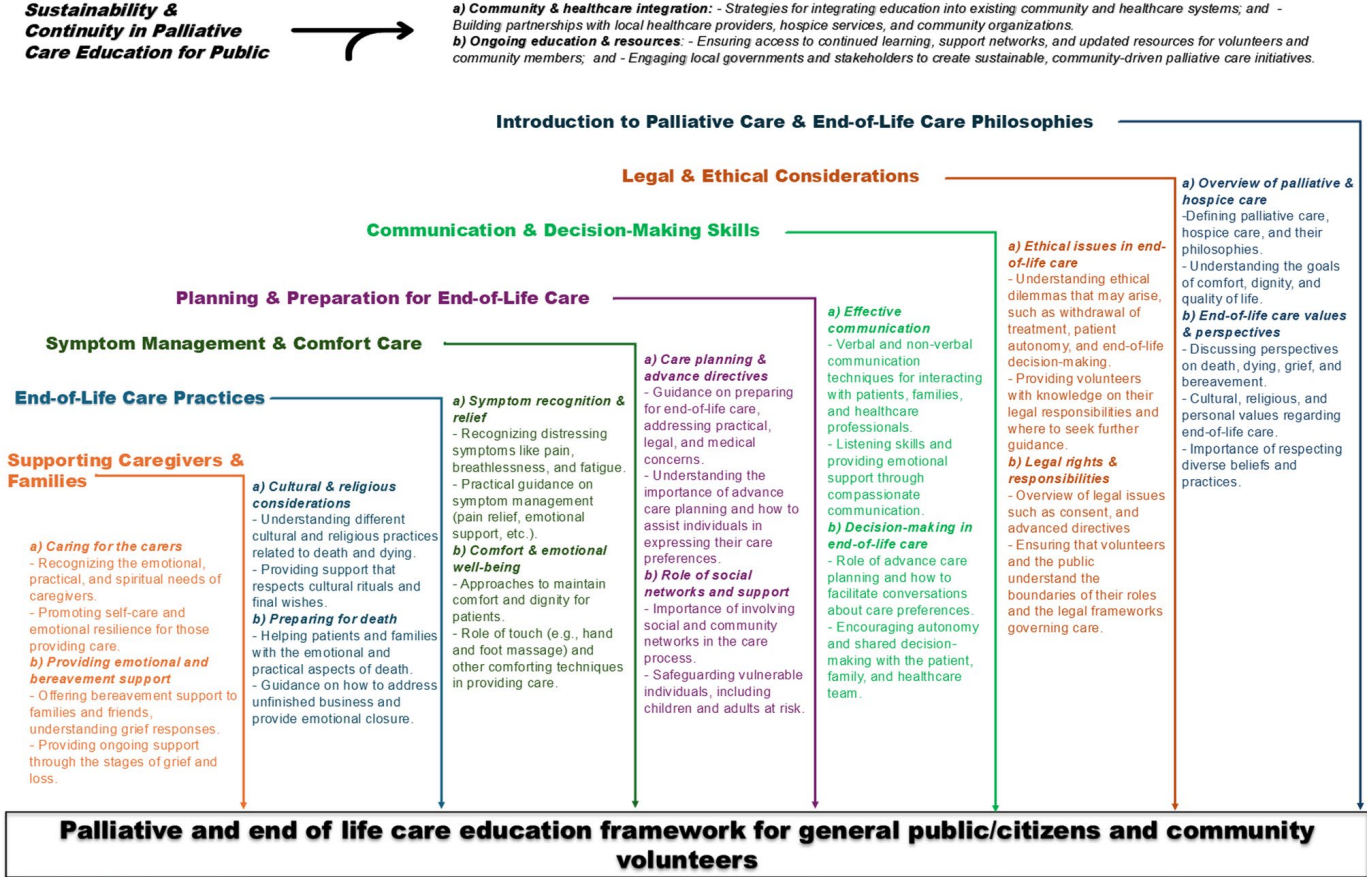
Palliative and end-of-life care education framework targeting the general public and community volunteers

Based on the findings of this review, we have developed a palliative and end-of-life care education framework targeting the general public and community volunteers, as illustrated in Fig. 3. From a palliative and supportive care perspective, providing plain language summaries of these domains may help the understanding of the care needs of individuals with palliative care requirements, as well as the needs of their family members. Figure 3 presents seven components, whereas the Results section outlines six. “Ethical and legal considerations” are included under foundational concepts and philosophies in the Results but presented separately in Fig. 3 to highlight their variability across national policies, legal frameworks, and cultural norms. We feel the additional component will be useful to those using the framework to highlight the local and national perspectives. Given the significant burden that long-term chronic conditions place on both patients and their families, any support and understanding offered by members of the community may potentially help to mitigate the associated physical and emotional distress [5, 9, 13].

Public health palliative care education could serve as a key mechanism to enable long-term follow-up for individuals wishing to remain at home during their final phase of life. Such education can foster robust social networks within communities, empowering individuals to support their neighbours [5, 13]. It is important to acknowledge that in certain circumstances, individuals may lack familial support during the palliative and end-of-life stages at home [41], particularly individuals in countries with low birth rates, where there may be fewer family members available to provide support, or where individuals may have no family members living nearby or at all. By promoting palliative and end-of-life care awareness within communities, these individuals may receive support from neighbours and close contacts, which could significantly improve both their quality of care and well-being [5, 9, 41]. Additionally, improving public understanding of palliative care can help ensure that individuals do not feel isolated or hopeless during the final stages of life. Widespread education in this domain could improve individuals’ ability to seek appropriate support, including knowledge of the institutions that provide such assistance. Therefore, we strongly recommend that public health policies incorporate the integration of palliative and end-of-life care education for the general population. Such policies should be prioritised by policymakers and embedded within healthcare systems to ensure the continuity of care and well-being for individuals with chronic conditions, alongside providing ongoing educational resources [5, 7, 9].

This review has several limitations that should be considered. It included studies published between 2013 and 2024 in the English language, which may have excluded contributions from countries with their own public palliative care education programmes or courses. Although we have aimed to create a comprehensive framework that accounts for cultural and social differences, this limitation may still have affected the review’s findings. As previously noted, few studies have examined the long-term effectiveness of this education in the community for patients and family caregivers. Therefore, further longitudinal studies are needed to understand and assess the effectiveness of these public health palliative care education programmes in practice and within communities and in improving long-term outcomes for both individuals receiving care and their families.

## Conclusion

The importance of community-based palliative and end-of-life care education for the general public and volunteers is unquestionable. This review identified six core components delivered to the public and volunteers through both in-person and online formats. Further research is needed to assess the long-term impact of these programmes in supporting individuals receiving palliative and end-of-life care and reducing the burden on healthcare systems. Delivering public health palliative education requires a collaborative approach, involving healthcare providers and community organisations. Developing strategies for integrating this education more effectively into healthcare systems and community networks is essential to ensure equitable access to quality palliative care.

## Electronic supplementary material

Below is the link to the electronic supplementary material.

Supplementary Material 1

## Data Availability

No datasets were generated or analysed during the current study.

## Abbreviations

EASE: End-of-Life Aid Skills for Everyone
PC: Palliative care
PEoLC: Palliative and end of life care
