# Supplementary material for: Key themes and approaches in palliative and end-of-life care education for the general public: a systematic review
Source: BMC Palliat Care. 2025 Aug 7;24:219. doi: 10.1186/s12904-025-01852-w (PMC12333110; doi:10.1186/s12904-025-01852-w)
Supplement: Supplementary file 1 — Supplementary Material 1 [file 12904_2025_1852_MOESM1_ESM.docx]

**Supplementary**

**Table 1** Medline search syntax

| Database: Ovid MEDLINE(R) ALL <1946 to February 01, 2024> Search Strategy: -------------------------------------------------------------------------------- 1     exp Palliative Care/ (64184) 2     exp Terminal Care/ (57741) 3     ((palliative or "end of life" or terminal or bereavement or grief or hospice) adj2 car*).ti,ab. (80586) 4     1 or 2 or 3 (153267) 5     exp Education/ (911309) 6     ("palliative care education*" or "education program*" or "end of life care education*").ti,ab. (35149) 7     5 or 6 (928977) 8     (public or famil* or communit* or citizen* or friend* or neighbour* or societ*).ti,ab. (2940222) 9     4 and 7 and 8 (2867) 10     limit 9 to (english language and yr="2013 -Current") (1357)  **************************************************************************** |
| --- |

**Table 2** Summary of the included studies’ critical appraisal using the appraisal tool by Hawker et al. (2002)

| Study | Abstract and title | Introduction and aims | Method and data | Sampling | Data analysis | Ethics | Bias | Results | Transferability or generalizability | Implications and usefulness | Total |
| --- | --- | --- | --- | --- | --- | --- | --- | --- | --- | --- | --- |
| Seymour 2013 | 4 | 4 | 3 | 3 | 3 | 3 | 3 | 4 | 3 | 3 | 33 |
| Pesut 2020 | 4 | 4 | 4 | 4 | 4 | 4 | 4 | 4 | 4 | 4 | 40 |
| Gerber 2013 | 2 | 4 | 3 | 2 | 1 | 1 | 1 | 3 | 2 | 4 | 23 |
| Burhansstipanov 2014 | 3 | 4 | 3 | 3 | 3 | 4 | 4 | 4 | 3 | 2 | 33 |
| Brighton 2017 | 4 | 4 | 4 | 4 | 4 | 4 | 4 | 4 | 3 | 3 | 38 |
| Hunter 2019 | 4 | 4 | 4 | 4 | 4 | 4 | 4 | 4 | 3 | 3 | 38 |
| Wang 2020 | 4 | 4 | 3 | 3 | 4 | 4 | 4 | 4 | 3 | 3 | 36 |
| Lee 2020 | 4 | 4 | 4 | 4 | 4 | 4 | 4 | 4 | 3 | 3 | 38 |
| Patterson 2022 | 3 | 4 | 4 | 4 | 4 | 2 | 2 | 3 | 4 | 3 | 33 |
| Aoun 2022 | 4 | 4 | 4 | 3 | 4 | 4 | 4 | 4 | 4 | 4 | 39 |
| Claxton-Oldfield 2022 | 4 | 4 | 4 | 3 | 3 | 4 | 4 | 3 | 3 | 3 | 35 |
| Pesut 2022 | 4 | 4 | 4 | 4 | 4 | 4 | 4 | 4 | 4 | 4 | 40 |
| Bollig 2021 | 4 | 4 | 4 | 4 | 4 | 4 | 4 | 4 | 4 | 3 | 39 |
| Aoun 2022 | 4 | 4 | 3 | 4 | 4 | 4 | 4 | 4 | 4 | 4 | 39 |
| Carter 2023 | 4 | 4 | 4 | 3 | 4 | 4 | 4 | 4 | 4 | 4 | 39 |
| Tieman 2018 | 4 | 4 | 4 |  | 4 | 4 | 4 | 4 | 4 | 4 | 36 |
| Miller-Lewis 2020 | 3 | 4 | 4 | 4 | 4 | 4 | 4 | 4 | 4 | 4 | 39 |
| Niinomi 2020 | 4 | 4 | 3 | 4 | 4 | 4 | 4 | 4 | 3 | 3 | 37 |
| Testoni 2021 | 4 | 4 | 4 | 4 | 4 | 4 | 4 | 4 | 4 | 3 | 39 |
| Bollig 2021 | 4 | 4 | 4 | 4 | 4 | 4 | 4 | 4 | 4 | 4 | 40 |

*Scoring items: 4=good, 3=fair, 2=poor, 1=very poor*

*Total scoring: good=40-31, fair=30-21, poor=20-11, very poor=10-1*
